# Editorial: The relationship of physical activity and cardiorespiratory fitness with acute COVID-19 infection and post COVID-19 conditions

**DOI:** 10.3389/fspor.2024.1444947

**Published:** 2024-07-22

**Authors:** J. Myers, A. W. Jones

**Affiliations:** ^1^ Division of Cardiology Veterans Affairs Palo Alto Health Care System, Palo Alto, CA, United States; ^2^ Department of Cardiology, Stanford University, Stanford, CA, United States; ^3^ Respiratory Research@Alfred, Monash University, Melbourne, VIC, Australia

**Keywords:** physical activity, cardiorespiratory fitness, COVID-19, physical function, cognitive function, systemic inflammation, immunity, infection

**Editorial on the Research Topic** Physical activity, cardiorespiratory fitness, respiratory infections, COVID-19, and “Long COVID”

Beginning in 2019, the world experienced an extraordinary, transformative challenge due to the COVID-19 pandemic. The pandemic revealed how a single highly infectious virus can overburden the healthcare systems of even economically developed nations (1, 2). Worldwide there has been >775 million reported cases and >7 million deaths from COVID-19^1^. The overall impact of COVID-19 took many additional forms, including physical, emotional, and economic. In addition to mortality, the consequences of ongoing illnesses, hospitalizations, long-term health complications, and loss of income or employment have been enormous, and far too challenging to quantify with precision.

One of the fundamental lessons that emerged from the COVID-19 pandemic was the fact that individuals with fewer cardiovascular disease (CVD) risk factors, healthier lifestyles, or both, had lower risks for severe COVID-19 outcomes, including mortality, admission to the ICU, and need for mechanical ventilation (3–5). In contrast, the most severe COVID-19 outcomes were most commonly associated with comorbidities that included obesity, hypertension, cardiovascular disease, smoking, and type 2 diabetes mellitus (3–6). A particular *health behavior* which has been associated with worse COVID-19 outcomes is physical inactivity (7). Potential mechanisms for better outcomes among individuals who acquired COVID-19 and were regularly physically active include better immune function, reduced systemic inflammation, improved cardiovascular health, improved muscle strength, and a better ability to withstand internal and external stressors, such as surgery or severe illness (8, 9). Overall, individuals who are more physically active or with greater cardiorespiratory fitness appear to be better equipped to withstand the physical and mental health challenges imposed by COVID-19 (3, 4, 8–10).

Cardiorespiratory fitness is in part a consequence of regular physical activity (11). In recent years, cardiorespiratory fitness has been demonstrated to be a powerful predictor of risk for mortality and other adverse health outcomes (4, 10–12). A growing body of research has demonstrated that higher levels of cardiorespiratory fitness reduce the risk of many highly prevalent noncommunicable diseases, including CVD, diabetes, and several site-specific cancers. The fact that cardiorespiratory fitness reflects the integrity of numerous systems at least partially explains the growing recognition that cardiorespiratory fitness predicts morbidity and mortality risk beyond commonly obtained risk factors. Higher cardiorespiratory fitness or regular moderate intensity physical activity has long been proposed to lower the risk of respiratory tract infections or improve vaccination responses due to immunomodulatory effects (13, 14). Skeletal muscle itself is recognized as an endocrine organ, whereby IL-6—the first coined “myokine”—is one of several immune mediators released upon muscle contraction and considered to be a key driver of the anti-inflammatory effects of regular physical activity (15). Such anti-inflammatory mechanisms underlie the benefits of high cardiorespiratory fitness and regular physical activity in lowering chronic low-grade inflammation or improving immune and inflammatory markers in several diseases including cancers, cardiovascular diseases, diabetes, and cognitive impairment (16, 17).

It has become clear from recent studies that lifestyle factors, including physical activity patterns and level of cardiorespiratory fitness, have had a clear relationship with health outcomes during acute COVID-19 infection and post COVID-19 conditions. This Research Topic of Frontiers in Sports and Active Living presents original research in these areas, including the impact of mild to moderate COVID-19 infection on cardiorespiratory fitness among firefighters. In those who were hospitalized with acute COVID-19 infection, this Research Topic reports the prevalence of systemic inflammation and low functional exercise capacity in people who self-reported symptoms with or without pulmonary lesions 6–12 months after hospital discharge. Furthermore, the physical and cognitive impairments with post-COVID-19 may be associated with reduced quality of life. We also learn that infection control measures such as lockdowns introduced in response to the COVID-19 pandemic affected mobility trends and resulted in a rise in population-level physical inactivity, particularly in older (ages 75+) and Black and Asian minority ethnicity (BAME) individuals. Clearly, an important public health lesson from the COVID-19 pandemic was that it provided yet another reason for promoting regular physical activity to the public. The COVID-19 pandemic led to trends in specific types of physical activity, and this Research Topic reports on a comparison of different group exercise formats whereby streaming and on demand group fitness are viable options for sustaining physical activity but physiological intensity and psychological perceptions may be greater during live class formats.

The articles in this Research Topic highlight the important role of physical activity and cardiorespiratory fitness during a global pandemic. Their findings will remain important with evolving SARS-CoV-2 variants and in preparedness for future viral pandemics, particularly in high-risk individuals.

## Footnotes

^1^World Health Organization Data Website, https://data.who.int/dashboards/covid19/data.

## References

[1] Filip R Gheorghita Puscaselu R Anchidin-Norocel L Dimian M Savage WK. Global challenges to public health care systems during the COVID-19 pandemic: a review of pandemic measures and problems. J Pers Med. (2022) 12(8):1295. 10.3390/jpm12081295 36013244 PMC9409667

[2] Kaye AD Okeagu CN Pham AD Silva RA Hurley JJ Arron BL Economic impact of COVID-19 pandemic on healthcare facilities and systems: international perspectives. Best Pract Res Clin Anaesthesiol. (2021) 35(3):293–306. 10.1016/j.bpa.2020.11.009 34511220 PMC7670225

[3] Arena R Lavie CJ, HL-PIVOT Network. The global path forward—healthy living for pandemic event protection (HL-PIVOT). Prog Cardiovasc Dis. (2021) 64:96–101. 10.1016/j.pcad.2020.05.008 32485186 PMC7834504

[4] Harber MP Peterman JE Imboden M Kaminsky L Ashton REM Arena R Cardiorespiratory fitness as a vital sign of CVD risk in the COVID-19 era. Prog Cardiovasc Dis. (2023) 76:44–8. 10.1016/j.pcad.2022.12.001 36539006 PMC9758758

[5] Wang S Li Y Yue Y Yuan C Kang JH Chavarro JE Adherence to healthy lifestyle prior to infection and risk of post-COVID-19 condition. JAMA Intern Med. (2023) 183(3):232–41. 10.1001/jamainternmed.2022.6555 36745445 PMC9989904

[6] Lange KW Nakamura Y. Lifestyle factors in the prevention of COVID-19. Glob Health J. (2020) 4(4):146–52. 10.1016/j.glohj.2020.11.002 33520339 PMC7834031

[7] Sallis R Young DR Tartof SY Sallis JF Sall J Li Q Physical inactivity is associated with a higher risk for severe COVID-19 outcomes: a study in 48,440 adult patients. Br J Sports Med. (2021) 55:1099–105. 10.1136/bjsports-2021-104080 33849909

[8] Nieman DC. Exercise is medicine for immune function: implications for COVID-19. Curr Sports Med Rep. (2021) 20(8):395–401. 10.1249/JSR.0000000000000867 34357885

[9] Arena R Hall G Laddu DR Phillips SA Lavie CJ. A tale of two pandemics revisited: physical inactivity, sedentary behavior and poor COVID-19 outcomes reside in the same syndemic city. Prog Cardiovasc Dis. (2022) 71:69–71. 10.1016/j.pcad.2021.11.012 34826425 PMC8616569

[10] Myers J Kokkinos P Cadenas-Sanchez C Liappis A Goraya NK Weintrob A The impact of cardiorespiratory fitness on COVID-19 related outcomes: the ETHOS study. Mayo Clinic Proceedings; (2024) (in press). 10.1016/j.mayocp.2024.07.004 39243247

[11] Ross R Blair SN Arena R Church TS Després JP Franklin BA Importance of assessing cardiorespiratory fitness in clinical practice: a case for fitness as a clinical vital sign. An American Heart Association scientific statement from the committee on physical activity and the council on lifestyle and cardiometabolic health. Circulation. (2016) 134:e653–99. 10.1161/CIR.0000000000000461 27881567

[12] Myers J McAuley P Lavie C Despres JP Arena R Kokkinos P. Physical activity and cardiorespiratory fitness as major markers of cardiovascular risk: their independent and interwoven importance to health status. Prog Cardiovasc Dis. (2015) 57:306–14. 10.1016/j.pcad.2014.09.011 25269064

[13] Nieman DC Henson DA Austin MD Sha W. Upper respiratory tract infection is reduced in physically fit and active adults. Br J Sports Med. (2011) 45(12):987–92. 10.1136/bjsm.2010.077875 21041243

[14] Woods JA Keylock KT Lowder T Vieira VJ Zelkovich W Dumich S Cardiovascular exercise training extends influenza vaccine seroprotection in sedentary older adults: the immune function intervention trial. J Am Geriatr Soc. (2009) 57(12):2183–91. 10.1111/j.1532-5415.2009.02563.x 20121985

[15] Pedersen BK. Exercise-induced myokines and their role in chronic diseases. Brain Behav Immun. (2011) 25(5):811–6. 10.1016/j.bbi.2011.02.010 21354469

[16] Pedersen BK Saltin B. Exercise as medicine—evidence for prescribing exercise as therapy in 26 different chronic diseases. Scand J Med Sci Sports. (2015) 25(Suppl 3):1–72. 10.1111/sms.12581 26606383

[17] Duggal NA Niemiro G Harridge SDR Simpson RJ Lord JM. Can physical activity ameliorate immunosenescence and thereby reduce age-related multi-morbidity? Nat Rev Immunol. (2019) 19(9):563–72. 10.1038/s41577-019-0177-9 31175337

